# CAF-specific markers: role of the TGFβ pathway

**DOI:** 10.18632/oncoscience.209

**Published:** 2015-08-21

**Authors:** Susann Busch, Göran Landberg

**Affiliations:** Sahlgrenska Cancer Center, Gothenburg University, Sweden

**Keywords:** breast cancer, fibroblast, TGFβ

The predominantly tumorigenic actions of transforming growth factor-beta (TGFβ) in aggressive cancer make the TGFβ pathway an attractive target for therapeutic intervention. Currently a number of TGFβ-targeted cancer therapies are in clinical trials. However, due to the multifaceted role of TGFβ signaling, antagonizing TGFβ in the clinical setting is challenging. Therefore it is essential to develop new predictive tests to account for subtype-specific treatment sensitivities, warranting optimization of more personalized therapeutic strategies.

TGFβ is a pleiotropic cytokine with potent anti-mitogenic and pro-apoptotic effects and is further regulating epithelial-mesenchymal transition (EMT), motility and cell differentiation. Thus, TGFβ signaling plays a key role at multiple steps during tumor progression including invasion, dissemination and metastatic colonization [1]. It is believed that during tumorigenesis a switch from anti-tumorigenic to tumor-promotive actions occurs [2]. However, response toward TGFβ is highly cell- and context-dependent and consequently in cancer, cellular functions mediated by TGFβ are complex. A TGFβ response signature has been associated with metastatic potential in estrogen receptor-alpha (ERα)-negative but not in ERα-positive breast cancer illustrating the need for cancer subtype dependent handling of TGFβ information [2].

Due to the dual function of TGFβ as a tumor-suppressor and tumor-promotor, it is paramount to fully elucidate context-dependent TGFβ-mediated actions and accordingly it is pivotal that the role of critical tumor microenvironmental factors such as the presence and role of cancer-activated fibroblasts (CAFs) is fully understood. TGFβ is a major player in the tumor-stromal crosstalk as it is a known key factor inducing transdifferentiation of fibroblasts into activated fibroblasts, a phenotype that has been linked to CAFs. We and others noted that stromal biomarkers possess clinical value such as CAF-specific SMAα expression which was linked to poor prognosis [3]. CAFs constitute the main component of the tumor microenvironment (stroma) and are typically characterized by a combination of mesenchymal markers such as smooth muscle actin-alpha (SMAα) and Vimentin. Classically CAFs are assigned with pro-tumorigenic qualities; however, recent advances have revealed tumor-inhibitory features. This suggests that tumor-residing fibroblasts exhibit some plasticity [4].

TGF-beta receptor type-2 (TGFBR2) is the sole ligand-binding receptor for all members of the TGFβ family and expressed in virtually all cell types including fibroblasts. It has been reported that blockade of the TGFβ pathway in mouse fibroblasts through conditional inactivation of the *Tgfbr2* gene is linked to increased oncogenic potential of the adjacent epithelia and consequently increased tumor formation and invasion, clearly demonstrating the tumor-suppressive role of fibroblastic TGFβ signaling [5, 6].

When analyzing clinical, *in vitro* and *in vivo* data, our studies collectively support the notion that TGFBR2 expression in CAFs is linked to prognostic features and key tumor properties in breast cancer [7]. We could further reveal the potential of fibroblasts to regulate cell growth as well as cell survival and further uncover a differential response of cancer cells in co-culture with normal, undifferentiated fibroblasts or with CAFs. Clonogenic (single-cell) survival seems to be dependent on the differentiation state of fibroblasts as monitored by SMAα expression. Blockade of TGFβ signaling in CAFs, either through application of a chemical inhibitor or shRNA-induced knockdown of TGFBR2, resulted in increased cell growth and survival of breast cancer cells. Overall, this study highlighted that the growth-inhibitory function of TGFβ pathway can be preserved in cancer. Further unpublished observations showed that using an equivalent setup of fibroblasts but co-cultured with ERα-negative breast cancer cells, cell growth was reduced upon TGFβ inhibition, signifying a switch to a more pro-tumorigenic role of TGFβ which was in contrast to the studies using ERα-positive breast cancer cells. Yet loss of fibroblastic TGFBR2 resulted in increased cell growth, underlining the prognostic value of CAF-specific TGFBR2 expression regardless of the ERα status.

**Figure 1:**
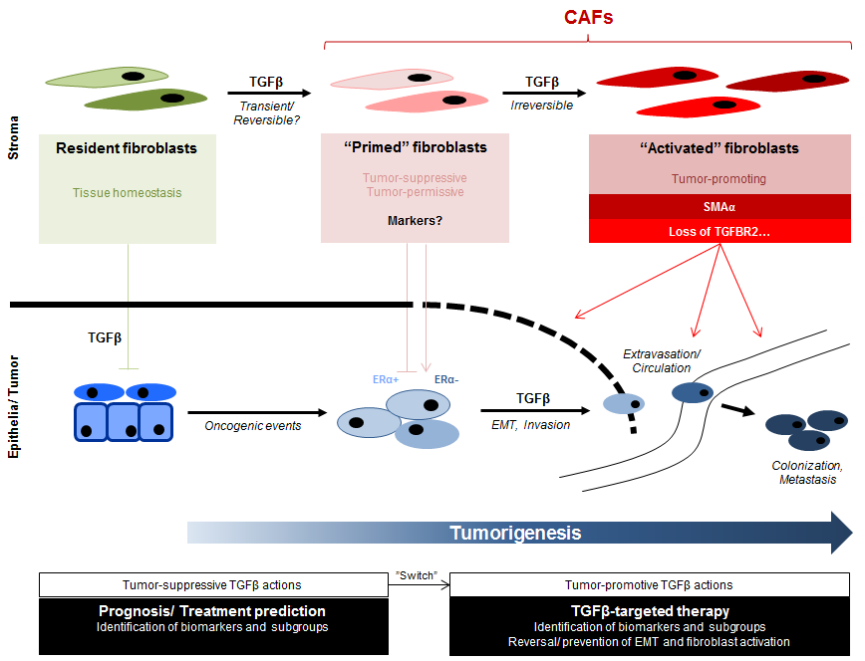
Role of TGFβ in the coevolution of tumor and stromal cells during tumor development Schematic diagram depicting multiple steps involving TGFβ during tumorigenesis. Cancer cells ultimately become refractory of TGFβ-mediated growth-inhibitory actions and TGFβ-induced differentiation of cancer-associated fibroblasts (CAFs) becomes increasingly evident, contributing to tumor progression. Origin of cell, oncogenic events and tumor-stromal crosstalk may contribute to observed heterogeneity within tumor and stromal compartment. Presence of distinct tumor-suppressive, -permissive or -promoting CAF subpopulations with different sets of phenotypic or functional markers need to be identified and further characterized. This will allow prediction of TGFβ response and thus applicability of TGFβ-targeted treatment strategies.

Although genetically stable, fibroblasts display a vast cellular heterogeneity and thus far no unique marker for CAFs has been identified. The existence of functional and phenotypic diverse fibroblast subpopulations may reflect either (1) different stages during fibroblast differentiation/activation, (2) different cells of origin (such as tissue-resident fibroblasts, mesenchymal stem cells, fibrocytes), (3) different modes of activation (eg. cancer subtype, chemokines, extra-cellular matrix), (4) a stochastic/hierarchical organization or (5) a combination of thereof. Ongoing studies aim now to define molecular subpopulations constituting fibroblast heterogeneity on single-cell level further enabling identification and characterization of desired reversal of pro-tumorigenic fibroblast activation states using pharmaceutical TGFβ inhibitors. A schematic overview of postulated function and evolution of CAF subpopulations is depicted in Figure 1.

CAFs as differentiated fibroblasts may operate with alternative pathways or altered signaling crosstalk, which is reflected in a distinct tumor-stromal interaction depending on the molecular profile of the cancer, emphasizing the importance to characterize tumor microenvironment subtypes. The impact of the tumor microenvironment on cancer growth and progression has become increasingly clinically relevant and should therefore be integrated in experimental designs to elucidate not only tumor-stromal interaction mechanisms but also drug-induced toxicity.
